# The Expression of Activation Markers CD25 and CD69 Increases during Biologic Treatment of Psoriasis

**DOI:** 10.3390/jcm12206573

**Published:** 2023-10-17

**Authors:** Michał Adamczyk, Joanna Bartosińska, Dorota Raczkiewicz, Małgorzata Kowal, Agata Surdacka, Danuta Krasowska, Anna Michalak-Stoma, Dorota Krasowska

**Affiliations:** 1 Department of Dermatology, Venereology and Pediatric Dermatology, Medical University of Lublin, 20-081 Lublin, Poland; jbartosinski@gmail.com (J.B.); kowalma71@o2.pl (M.K.); annamichalak@wp.pl (A.M.-S.); dor.krasowska@gmail.com (D.K.); 2 Department of Cosmetology and Aesthetic Dermatology, Medical University of Lublin, 20-093 Lublin, Poland; 3 Department of Medical Statistics, Center of Postgraduate Medical Education, School of Public Health, 01-826 Warsaw, Poland; dorota.raczkiewicz@cmkp.edu.pl; 4 Department of Clinical Immunology, Medical University of Lublin, 20-093 Lublin, Poland; agatasurdacka@op.pl; 5 Department of Medical Chemistry, Doctoral School, Medical University of Lublin, 20-093 Lublin, Poland; dana.krasowska@gmail.com

**Keywords:** psoriasis, systemic treatment, biologic treatment, activation markers

## Abstract

CD (cluster of differentiation) 69 and CD25 are considered early and late markers of the activation of lymphocytes, respectively. CD25 is a part of the IL-2 receptor and is present on the surface of immune and non-immune cells, with high amounts on activated lymphocytes and regulatory T cells. CD69 is expressed on various types of white blood cells, including newly activated lymphocytes, lymphocytes infiltrating tissues isolated from subjects with chronic auto-inflammatory diseases, several subtypes of memory T cells and regulatory T cells. Primarily, CD69 was considered to be an early marker of the activation of lymphocytes, but, right now, data derived from in vitro and in vivo studies have revealed the immunomodulatory role of this surface antigen. In 84 patients with psoriasis, of whom 28 were treated with different biologic drugs, as well as in 29 healthy control subjects, the expression of CD25 and CD69 on different subtypes of peripheral blood mononuclear cells (PBMCs) was studied with the use of flow cytometry. Significantly higher levels of CD3/CD69-, CD8/CD69- and CD19/CD69-positive PBMCs as well as within CD3+ cells were present in subjects suffering from psoriasis when compared to healthy controls. In patients with psoriasis who were treated with biologic drugs, the levels of CD3/CD69-, CD4/CD69- and CD19/CD69-positive PBMCs, and CD3/CD69 within CD3+ cells, CD4/CD69 within CD4+ cells, CD4/CD25 within CD4+ cells and CD19/CD69 within CD19+ cells were significantly higher than before therapy. Our results support a role for activation markers, especially CD69, in psoriasis. Further research is warranted to fully clarify their significance in this common dermatosis, especially during biologic treatment.

## 1. Introduction

Psoriasis vulgaris is a common chronic skin disease, manifesting with clinically typical erythematous papules and plaques with silvery white scales on their surface. As an immune-mediated condition associated with immune system imbalance, psoriasis is accompanied by a wide spectrum of possible comorbidities, and, today, it is treated as a systemic disease rather than solely a skin problem. The appearance of skin lesions results from a complex relationship between environmental and genetic factors and is due to the activation, proliferation and infiltration of the dermis with Th1 and Th17 cells, with a secondary inflammatory reaction and massive hyper-proliferation of keratinocytes [1]. However, in psoriasis, the impairment in normal function concerns the majority of immune and non-immune cells, including but not limited to dendritic cells, monocytes, macrophages, mast cells, neutrophils, keratinocytes and natural killer (NK) cells [2]. Despite the large amount of research, the exact pathogenesis of psoriasis remains unclear. Nevertheless, recent advances in our understanding of immune system dysregulation in this disease have led to the development of many different biologic drugs, targeting crucial cytokines and/or receptors involved and enabling great treatment response, changing the outcomes in this tenacious ailment [3].

Clusters of differentiation (CD)25 and CD69 are considered markers of the activation of lymphocytes; however, to consider them only in this way is a gross oversimplification. CD25 is a part of the interleukin (IL)-2 receptor and is expressed on the surface of both immune and non-immune cells, with high amounts on activated lymphocytes and regulatory T cells [4]. CD25 inhibitory drugs, basiliximab and daclizumab, prevent the activation and proliferation of lymphocytes mediated by IL-2, and they are used as immunosuppressants in the prevention of graft rejection after organ transplantations [5]. In the context of psoriasis, CD25 has mainly been described as a T-regulatory lymphocyte (T_reg_) marker. A defect in the proper function of CD4+CD25+ forkhead box protein 3 (Foxp3)+ T_regs_ has been reported in this group of patients [6].

CD69 is a transmembrane type II lectin protein that appears early on the surface of lymphocytes after stimulation; however, its amounts drop several hours later. This antigen is also detectable in negligible quantities on the surface of resting lymphocytes and in steady states on T_regs_ and some subtypes of memory cells [7]. Research data show that CD69 may exert immunomodulatory effects, playing a crucial role in the development of immune-mediated conditions. For example, it has been indicated that CD69 may stimulate the suppressive properties of T_regs_ and control the balance between the differentiation of T helpers (Ths) and T_regs_. On the contrary, some human studies have shown that CD69 may also have a stimulatory effect on the inflammatory response [7].

Literature data concerning the role of CD25 and CD69 in psoriasis are quite sparse and require expansion. In this study, we evaluated the expression of the activation markers CD25 and CD69 on peripheral blood mononuclear cells (PBMCs) in subjects with psoriasis and healthy individuals. In a subset of psoriatic patients, analyses were repeated after biologic treatment, with several classes of monoclonal antibodies approved in this indication.

## 2. Materials and Methods

### 2.1. The Study Group

The current study comprised 84 patients with chronic plague psoriasis who were under care of Department of Dermatology, Venerology and Pediatric Dermatology, Medical University of Lublin, Poland. Inclusion criteria were as follows: age ≥ 18 years and active psoriatic skin lesions of more than 1-year duration. Exclusion criteria included the following: other psoriasis of different types (i.e., erythrodermic, guttate and pustular), concurrent immune-mediated diseases, malignancies and currently taking systemic treatment for psoriasis and other immunomodulatory/immunosuppressive agents. The control group included 29 subjects without psoriasis and different immune-mediated ailments. In 28 subjects with psoriasis, blood samples were collected twice: before and during treatment with biologic drugs (no sooner than 16 weeks after start of the therapy).

Table 1 presents detailed clinical characteristics of the studied population.

In a subset of patients who were treated with biologics, the following medicaments were used: adalimumab (12 subjects), secukinumab (6 subjects), ustekinumab (5 subjects), ixekizumab (3 subjects), risankizumab (1 subject) and infliximab (1 subject). All patients who were qualified to undergo biologic therapy satisfied all of the following requirements: baseline PASI score > 18, BSA > 10 and DLQI > 10; furthermore, they must have been treated before with at least two standard systemic treatments (including methotrexate, cyclosporine, acitretin and PUVA phototherapy) without satisfactory response or experiencing intolerance to them. The requirements described above are in line with the rules for qualifying patients for reimbursable biologic drug therapy in Poland. For all subjects qualified to take part in the study, the biologic drug given here was their first biologic treatment. The dosing regimens for each drug were standard, as approved in drug characteristics (no dose optimization was required).

The approval for the study was given by Bioethics Committee of Medical University of Lublin (decision no: KE-0254/165/2017). We took written informed consent from all study subjects before participation in the study. Current research included the same group of psoriatic patients and control group that was published recently [8].

### 2.2. Evaluation of CD25 and CD69

To start, 30 mL of peripheral blood was collected from the ulnar vein into tubes containing EDTA. Immediately after collection, the samples were incubated at room temperature in the dark for 20 min. Then, we added 2 mL of BD FACS lysing solution (BD Pharmingen, San Diego, CA, USA) to the tubes. We isolated PBMCs by using density gradient centrifugation on Ficoll–Hypaque (Biochrom AG, Berlin, Germany). We removed interphase cells and washed them twice in PBS (phosphate-buffered saline) without Ca^2+^ and Mg^2+^. Prepared cells were then suspended in Roswell Park Memorial Institute (RPMI) 1640 medium with 2% human albumin. Tryptan blue staining was used to evaluate the viability of obtained PBMCs. Samples with cell viability of less than 90% were disqualified from subsequent testing. We used Neubauer chamber to quantify viable cells and incubated 5 × 10^5^ cells at room temperature for 20 min with monoclonal antibodies (mAbs) labelled with fluorochrome.

The set of mAbs and fluorochromes utilized is presented in Table 2. The immunophenotype of PBMCs was assessed using a FACSCalibur flow cytometer (Becton Dickinson, Franklin Lakes, NJ, USA) containing an argon laser (488 nm of wavelength). CellQuest software (version 5.1, Becton Dickinson, USA) was then used to perform acquisition and analysis of results. To optimize the flow cytometer settings, we applied the CaliBRITE calibration kit (Becton Dickinson, USA).

The following combinations of antibodies were added to cytometric tubes:

1. Anti-CD3 FITC/anti-CD25 PE/anti-CD69 PE;

2. Anti-CD4 FITC/anti-CD25 PE/anti CD69 PE;

3. Anti-CD8 FITC/anti-CD25 PE/anti CD69 PE;

4. Anti-CD19 FITC/anti-CD25 PE/anti CD69 PE.

**Figure 1:**
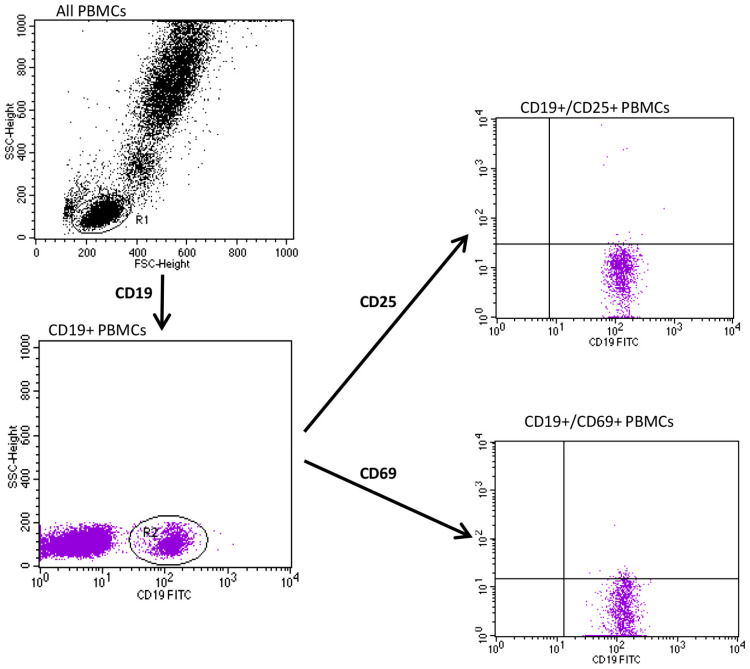
The gating strategy for flow cytometry evaluation of CD25 and CD69 expression on CD19+ PBMCs in subjects with psoriasis.

**Figure 2:**
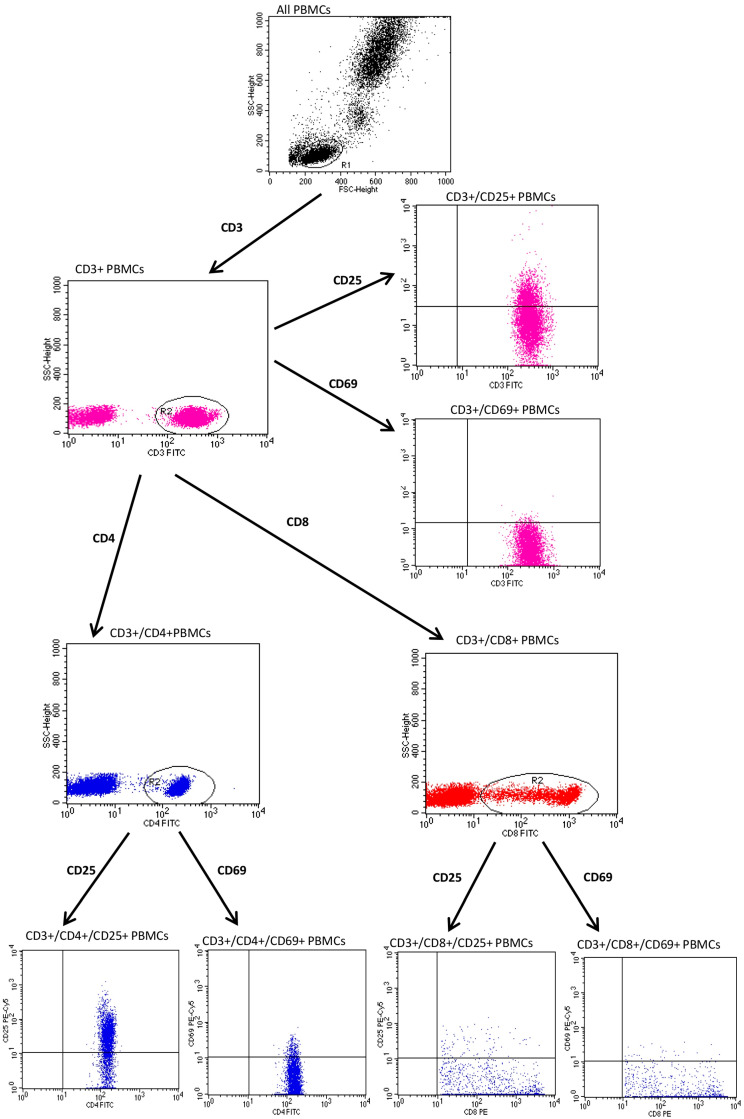
The gating strategy for flow cytometry evaluation of CD25 and CD69 expression on CD3+ PBMCs in subjects with psoriasis.

To identify and gate lymphocytes, we set adequate forward and side parameters. The gating strategy for cytometric studies of CD25 and CD69 expression on PBMCs from subjects with psoriasis is shown in Figure 1 and Figure 2.

**Figure 3:**
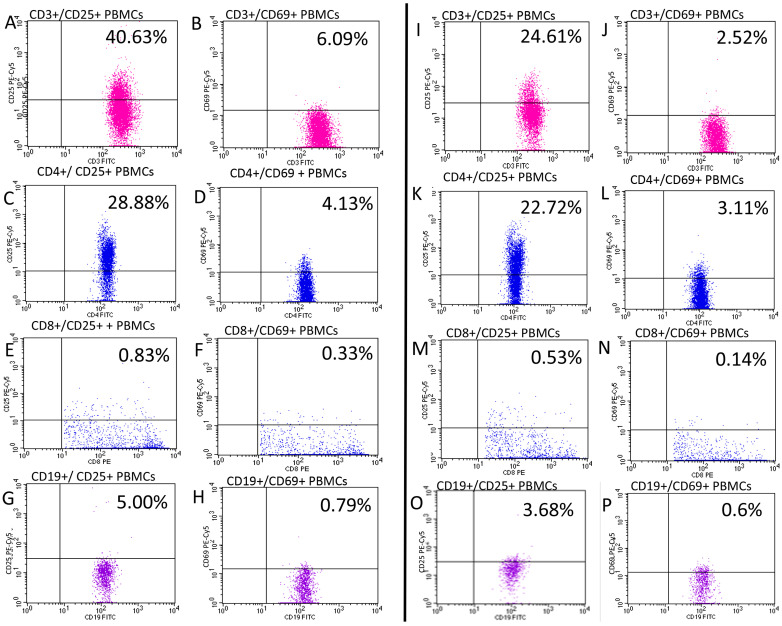
Illustrative flow cytometry assessment of the expression of CD25 (**A**,**C**,**E**,**G**,**I**,**K**,**M**,**O**) and CD69 (**B**,**D**,**F**,**H**,**J**,**L**,**N**,**P**) in patient with psoriasis (**A**–**H**) and healthy control (**I**–**P**). Pink color—CD3+ T-cells, blue—CD4+ T-cells, red—CD8+ t-cells and purple—CD19+ cells.

Cytometric assessment results were shown as the percentage rate of cells stained with fluorescent dye-conjugated monoclonal antibodies and as mean fluorescence intensity (MFI), which is an exponent of the amount of expression of a given antigen on the cell surface. Figure 3 presents illustrative flow cytometry assessment of CD25 and CD69 expression in patients with psoriasis and healthy controls.

### 2.3. Statistical Methods

We used STATISTICA 13.1 software (STATSOFT, Cracow, Poland) for statistical analyses of the obtained results. Median and interquartile ranges (IQRs) for continuous variables or absolute numbers (n) and relative numbers (%) of occurrence of items of categorical variables were estimated. In all the statistical tests, results were interpreted as statistically significant when *p*-value was less than 0.05.

We used U Mann–Whitney test for comparison of age and percentage rates of each PBMC subset between subjects with psoriasis and control group. In psoriatic patients, before and during biologic treatment, Wilcoxon test was applied to compare differences between levels of each PBMC subset. Correlation between CD25 and CD69 expression on different PBMCs with PASI, BSA and duration of psoriasis was evaluated using Spearman correlation coefficient.

## 3. Results

Significantly higher concentrations of CD3/CD69-, CD8/CD69- and CD19/CD69-positive PBMCs were shown in patients with psoriasis when compared to the healthy control group (Table 3).

In subjects with psoriasis who were treated with biologic drugs, the levels of CD3/CD69-, CD4/CD69- and CD19/CD69-positive PBMCs as well as CD3/CD69 within CD3+ cells, CD4/CD69 within CD4+ cells, CD4/CD25 within CD4+ cells and CD19/CD69 within CD19+ cells were significantly higher than before therapy (Table 4).

Regarding PASI, BSA and the duration of psoriasis, we found a positive correlation between the expression of CD4/CD69 within CD4 and PASI: the more severe the psoriasis was, as assessed via PASI, the higher percentages of CD4/CD69 within CD4+ lymphocytes. A positive correlation between the expression of CD3/CD25 within CD3+ cells and the duration of psoriasis was revealed: the longer the duration was, the higher the percentage of CD3/CD25 within CD3+ cells (Table 5).

## 4. Discussion

CD69 and CD25 are considered early and late markers of the activation of immune cells, respectively. To date, very little research has focused on their role in the pathomechanism of psoriasis; however, data show that they may play an important role in this disease, as well as in many other immune-mediated ailments.

In terms of CD25, as a part of the IL-2 receptor, it plays a critical role in proper immune system function, especially by conducting activation signals in lymphocytes mediated by IL-2 but also being an important marker of T_regs_, which play a major role in maintaining immunotolerance and inhibiting the pathological activation of immune cells and, thus, preventing the development of immune-mediated diseases. At the turn of the millennium, there were attempts made to treat patients with severe psoriasis with biologic drugs targeting CD25. However, due to potent immunosuppressive effects, these drugs were not proceeded for this indication [9].

These days, in the context of CD25 and psoriasis, the majority of research is focused on T_regs_ and their function. Accumulating data show that the impaired function of T_regs_ contributes to the development of psoriasis. In particular, IL-23 is a key cytokine involved in the stimuli conversion of T_regs_ into T-helper (Th)17 cells, producing large amounts of IL-17A. Furthermore, IL-17A reduces T_regs_ activity by decreasing transforming growth factor beta (TGFβ) and Foxp3 [6]. In a murine model of psoriasis, isolated CD4+CD25+ T_regs_ showed decreased cell proliferation abilities and a weakened immunosuppressive function, with significantly increased levels of PI3K pathway effectors, pAKT and pFoxo1 [10]. The dysregulated Akt-Foxo1 pathway was described in T_regs_ in a murine model of psoriasis, and it is thought to be a critical factor contributing to the impaired function of these cells [11].

Studies regarding the levels and function of T_regs_ in PsA have produced controversial results. In a recently published meta-analysis, the authors showed decreased levels of several subtypes of T_regs_ in this group of patients, in particular CD4+ CD25+FoxP3+ T_regs_ and CD4+CD25highCD127low T_regs_. Subjects with PsA also had lower levels of transforming growth factor beta (TGFβ) [12].

Of great importance, the therapeutic agents currently used in the treatment of psoriasis, including retinoids, vitamin D3 analogues, dimethyl fumarate, narrow-band UVB and biologic drugs targeting subunit p19 of IL-23 and IL-17A, all have the effect of restoring the defected T_regs_ [6]. An interesting study performed by Mehta et al. compared the effect of guselkumab and secukinumab treatment on t-cell profiles within psoriatic skin lesions. They found that only secukinumab therapy resulted in an increase in CD4+CD25+FoxP3+ T_regs_, whereas guselkumab did not have any impact [13]. This may be due to the direct impact of IL-17A on the conversion of T regs to Th17cells, as described above [6]. Quaglino et al. demonstrated that an increase in the number of T_regs_ during biologic therapy was positively correlated with response to treatment [14].

Many research studies showed an elevated expression of CD25 on the surface of circulating PBMCs in subjects with psoriasis [15,16]. Several of them evaluated the impact of cyclosporine on the amounts of circulating PBMCs expressing CD25. Economidou et al. found increased baseline CD3+CD25+ T cells and a reduction in CD25 expression after treatment with cyclosporine [17]. Similarly, Langewouters et al. demonstrated a significant decrease in CD4+CD25+ T cells soon after the introduction of treatment with afefacept [18]. Reddy et al. evaluated the expression of some subtypes of T cells before and during therapy with ustekinumab and found no impact of this therapy on the levels of circulating lymphocytes expressing CD25 [19]. Additionally, in vitro studies demonstrated that activated CD4+CD25+ T cells were significantly reduced by adalimumab, ixekizumab and tocilizumab in PsA patients as compared to a medium [20]. Our study did not reveal differences in the expression of CD25 between subjects with psoriasis and a healthy control group (Table 3). Biologic treatment resulted in a slight increase in CD4+CD25+ PBMCs but only within CD4 cells, solely, with no impact on other subtypes of CD25+ PBMCs (Table 4). This result may be due to the restorative effect of biologic treatment on T_regs_, as described above.

While discussing CD69 and psoriasis, this molecule has been shown to be a tissue-resident memory T cell (TRM) marker, together with CD8 and CD103. This subset of cells plays an important physiological role in the defense against infectious agents invading the skin; however, their pathological activation plays an important role in the development of immune-mediated skin diseases, i.e., psoriasis and vitiligo. The presence of TRM cells is responsible for recurrences of skin symptoms after the cessation of therapy [21]. Even more important findings demonstrated that CD69 on the skin Tγδ cells controls the secretion of IL-22 by regulating the uptake of L-tryptophan by aromatic amino acid transporter complex LAT1-CD98, which contributes to the development of psoriasis induced by IL-23. The same mechanisms were shown in circulating Vγ9 γδ T cells [22].

Research performed by Kim et al. showed that the expression of CD69, as well as CTLA4 and PD-L1, is higher in skin lesion biopsies taken from subjects with mild psoriasis when compared to those with severe forms of disease [23]. In subjects with psoriatic arthritis, increased levels of CD8+ CCR6+ T-cell effectors expressing CD69 were found in peripheral blood, and the accumulation of CXCR3+ CD8+ and CD69+T cells was also revealed in the synovial fluid of inflamed joints [24]. Conversely, Cameron et al. revealed that the amount of circulating, activated CD69+ lymphocytes was not elevated in psoriatic patients when compared to a control group. However, their research included a limited sample of fourteen subjects only and evaluated all PBMCs expressing CD69 [25].

Our results showed significantly higher levels of CD3/CD69-, CD8/CD69- and CD19/CD69-positive PBMCs as well as within CD3+ cells present in patients with psoriasis when compared to the healthy control group, which is in agreement with the above findings (Table 3). As indicated, CD69 may exert ambiguous effects on the immune response, having been shown to enhance immune system activation but also controlling the balance between Th/T_reg_ cells and stimulating the suppressive activity of T_regs_ [7].

Some therapies and/or procedures have been shown to impact CD69 expression in subjects with psoriasis. It has been shown that the in vitro exposure of lymphocytes to ultraviolet B (UVB) light results in a reduction in early and late markers of activation, including CD69 and CD25, respectively, together with many other inflammatory receptors and molecules, including [26].

Gaál et al. evaluated the effect of systemic alphacalcidol on several immunologic aspects in subjects with psoriasis and revealed that this intervention resulted in a statistically significant decrease in CD3+/CD69+ PBMCs, together with a continuous decrease in symptoms of the disease [27]. An interesting study was published by Haider et al., in which CD69 was identified as a potential marker for non-responders to therapy with alefacept, an anti-CD2 biologic drug. They revealed a higher expression of CD69 in PBMCs before treatment in non-responders to treatment than in responders. Alefacept was finally withdrawn from the market due to poor efficacy for psoriasis [28]. In another study evaluating the impact of anti-psoriatic treatment on immune system functioning in psoriatic patients, Rubant et al. revealed that dimethylfumarate caused a dose-dependent reduction in the expression of CD25 and slightly increased the expression of CD69 [29].

Several studies evaluated the impact of anti-psoriatic treatments on the expression of activation markers in skin samples. For example, Torres-Álvarez et al. studied the effect of methotrexate treatment on the expression of cell adhesion molecules and CD69 in subjects with psoriasis and found no impact of this treatment on the amounts of CD8+CD69+ T cells [30]. Similarly, Sigmundsgottir et al. found that in psoriatic subjects, treatment with UVB phototherapy did not result in any change in the expression of the activation markers CD25 and CD69 on PBMCs [31].

A recently published study evaluated the influence of methotrexate and biologic treatment with different agents (including adalimumab, secukinumab and ixekizumab) on TRM in psoriatic lesions. The authors demonstrated a decrease in the expression of TRM markers, including CD69 after 4 and 12 weeks of therapy in all treatments, with the most rapid response in subjects treated with anti-IL-17 agents. Interestingly, this decrease was more pronounced in the dermis when compared to the epidermis, which may be due to the poorer penetration of systemic agents to the epidermis [32].

In the present study, after biologic therapy, there were significantly higher levels of several subtypes of lymphocytes expressing CD69 (CD3/CD69-, CD4/CD6-9 and CD19/CD69-positive PBMCs as well as CD3/CD69 within CD3+ cells, CD4/CD69 within CD4+ cells and CD19/CD69 within CD19+) (Table 4). These findings support the role of CD69 in psoriasis, as elaborated above, and the influence of biologic treatment on the expression of this antigen.

In our work, we performed statistical analysis of CD69 and CD25 expression on different PBMCs with PASI, BSA and the duration of psoriasis, but we found only a few correlations (Table 5). Their clinical significance is difficult to assess.

The limitations of the present study include the relatively small amount of subjects with psoriasis recruited undergoing biologic therapy. The expression of CD25 and CD69 was performed in peripheral blood only and not in the lesional skin. Due to the limited number of subjects taking different biologic drugs, we did not analyze them separately depending on the drug received.

## 5. Conclusions

Our results and data from the literature support the role of the activation markers CD25 and CD69 on PBMCs in psoriasis. The expression of CD69 within CD4+ T cells is positively correlated with the severity of skin lesions. Biologic treatment positively impacts the expression of some subsets of CD69-positive PBMCs, which may stimulate the suppressive properties of T_regs_ and be one of the reasons for their great efficacy. Further research on larger populations is warranted to clarify their exact role in psoriasis and the clinical significance of these findings.

## Figures and Tables

**Table 1:** Characteristics of the study patients with psoriasis and healthy control group.

Characteristics	Unit or Category	Results
**Patients with psoriasis (N = 84):**		
Age, min–max, Median (IQR)	Years	18–68, 43 (34–52)
Gender, n (%)	Male	60 (71.4)
	Female	24 (28.6)
Weight, min–max, Median (IQR)	kg	47–125, 85 (74–95)
BMI, min–max, Median (IQR)	kg/m^2^	17.72–46.88, 27.51 (24.44–31.25)
Smoking status, n (%)	Non-smokers	30 (38.0)
	Smokers	54 (62.0)
Psoriasis type, n (%) *	I	72 (88.1)
	II	12 (11.9)
Duration of psoriasis, min–max, Median (IQR)	Years	1–50, 20 (14–26)
PASI, min–max, Median (IQR)		5.5–47.0, 18.0 (13.1–21.3)
BSA, min–max, Median (IQR)		6–80.0, 23.3 (15.0–39.8)
PsA, n (%)	Yes	27 (30.9)
**Control group (N = 29):**		
Age, min-max, Median (IQR)	Years	24–65, 40 (33–52)
Gender, n (%)	Male	20 (69.0)
	Female	9 (31.0)

IQR—interquartile range, * type I: onset before age of 40, type II: onset after age of 40.

**Table 2:** Set of monoclonal antibodies (mAbs) and fluorochromes applied to analyze the expression of peripheral blood mononuclear cells (PBMCs) via surface antigen analysis.

Specificity	Fluorochrome	Producer	Clone	Isotype
Mouse anti human-**CD3**	FITC	BD Biosciences, USA	MEM-57	Mouse IgG2a, κ
Mouse anti human-**CD4**	FITC	BD Biosciences, USA	RPA-T4	Mouse IgG1, κ
Mouse anti human-**CD8**	FITC	BD Biosciences, USA	SK1	Mouse BALB/c IgG1, κ
Mouse anti human-**CD19**	FITC	BD Biosciences, USA	HIB19	Mouse IgG1, κ
Mouse anti human-**CD25**	PE-Cy^TM^5	BD Biosciences, USA	M-A251	Mouse BALB/c IgG1, κ
Mouse anti human-**CD69**	PE-Cy^TM^5	BD Biosciences, USA	FN50	Mouse IgG1, κ

**Table 3:** A comparison of percentage rates of PBMCs expressing CD69 and CD25 between subjects with psoriasis (N = 84) and healthy controls (N = 29).

PBMC Subtype	Psoriasis	Control	*p*
	Median (IQR)	Median (IQR)	
CD3/CD69	3.22 (2.18–5.81)	2.07 (1.68–3.00)	**0.006**
CD3/CD69 within CD3	4.19 (2.87–7.75)	3.17 (2.33–4.40)	**0.018**
CD3/CD25	27.97 (21.87–33.59)	24.94 (21.41–29.00)	0.107
CD3/CD25 within CD3	37.74 (30.43–44.68)	35.95 (31.00–41.88)	0.349
CD4/CD69	2.13 (1.27–3.59)	1.80 (1.36–2.13)	0.071
CD4/CD69 within CD4	4.49 (2.85–7.84)	3.61 (2.92–5.25)	0.090
CD4/CD25	23.74 (18.83–28.79)	22.88 (18.36–27.27)	0.740
CD4/CD25 within CD4	56.53 (47.77–64.50)	54.20 (50.50–57.08)	0.363
CD8/CD69	0.27 (0.16–0.55)	0.22 (0.14–0.26)	**0.017**
CD8/CD69 within CD8	1.09 (0.55–1.78)	0.53 (0.32–0.73)	**<0.001**
CD8/CD25	0.57 (0.37–0.97)	0.54 (0.43–0.82)	0.723
CD8/CD25 within CD8	1.86 (1.27–3.34)	1.56 (1.12–2.20)	0.210
CD19/CD69	0.66 (0.32–1.13)	0.42 (0.28–0.60)	**0.035**
CD19/CD69 within CD19	8.08 (3.99–12.54)	6.50 (3.14–9.90)	**0.068**
CD19/CD25	1.88 (1.27–2.67)	1.98 (1.28–2.43)	0.989
CD19/CD25 within CD19	23.49 (16.26–31.38)	24.91 (19.41–29.30)	0.404

*p* for U Mann–Whitney test, IQR—interquartile range. Statistically significant correlations in bold.

**Table 4:** A comparison of percentage rates of PBMCs expressing CD69 and CD25 in subjects with psoriasis treated with biologic drugs before and during treatment (N = 28).

PBMC Subtype	Before Treatment	During Treatment	*p*
	Median (IQR)	Median (IQR)	
CD3/CD69	2.86 (1.69–3.68)	3.73 (1.61–18.83)	**0.052**
CD3/CD69 within CD3	3.74 (2.36–4.79)	5.01 (2.16–37.84)	**0.044**
CD3/CD25	25.15 (22.99–34.79)	29.26 (22.27–33.98)	0.755
CD3/CD25 within CD3	37.09 (33.28–44.26)	41.75 (31.87–45.95)	0.280
CD4/CD69	1.69 (0.90–2.43)	2.87 (1.02–11.70)	**0.019**
CD4/CD69 within CD4	3.80 (2.25–5.68)	7.16 (2.60–42.45)	**0.005**
CD4/CD25	23.53 (18.05–32.33)	25.11 (21.41–29.50)	0.471
CD4/CD25 within CD4	55.33 (49.03–66.15)	59.65 (51.83–66.83)	**0.041**
CD8/CD69	0.21 (0.15–0.36)	0.27 (0.11–5.72)	0.094
CD8/CD69 z CD8	0.94 (0.43–1.28)	1.06 (0.34–18.06)	0.073
CD8/CD25	0.57 (0.42–0.97)	0.73 (0.43–0.98)	0.638
CD8/CD25 z CD8	2.15 (1.35–2.76)	2.40 (1.23–3.10)	0.981
CD19/CD69	0.39 (0.28–0.77)	0.99 (0.19–4.80)	**0.004**
CD19/CD69 within CD19	6.16 (2.78–9.82)	9.40 (2.58–45.89)	**0.006**
CD19/CD25	1.94 (1.20–2.83)	2.34 (1.33–3.66)	0.088
CD19/CD25 within CD19	24.42 (16.10–30.66)	24.46 (15.53–33.06)	0.220

*p* for Wilcoxon rank test, IQR—interquartile range. Statistically significant correlations in bold.

**Table 5:** Correlations of the expression of CD69 and CD25 on different PBMC subsets with PASI, BSA and duration of psoriasis (N = 84).

PBMC Subtype	PASI		BSA		Duration of Psoriasis	
	r	*p*	r	*p*	r	*p*
CD3/CD69	0.178	0.105	−0.031	0.776	−0.046	0.682
CD3/CD69 within CD3	0.174	0.113	−0.051	0.643	−0.030	0.788
CD3/CD25	−0.063	0.576	0.143	0.200	0.122	0.280
CD3/CD25 within CD3	−0.138	0.209	0.051	0.648	0.224	**0.043**
CD4/CD69	0.188	0.087	−0.038	0.732	−0.059	0.599
CD4/CD69 within CD4	0.221	**0.043**	−0.013	0.909	−0.083	0.457
CD4/CD25	−0.094	0.397	0.063	0.572	0.043	0.702
CD4/CD25 within CD4	−0.157	0.154	−0.019	0.867	0.144	0.197
CD8/CD69	0.133	0.228	−0.172	0.118	−0.047	0.674
CD8/CD69 within CD8	0.101	0.363	−0.147	0.183	0.001	0.990
CD8/CD25	−0.070	0.530	−0.032	0.771	0.039	0.725
CD8/CD25 within CD8	−0.145	0.189	−0.068	0.540	0.055	0.625
CD19/CD69	0.213	**0.052**	0.015	0.891	−0.144	0.310
CD19/CD69 within CD19	0.125	0.259	−0.033	0.766	−0.147	0.188
CD19/CD25	0.069	0.533	0.043	0.702	0.033	0.771
CD19/CD25 within CD19	−0.010	0.927	−0.003	0.979	0.013	0.907

r—Spearman correlation coefficient, statistically significant correlations in bold.

## Data Availability

All data presented in this study are reported in this manuscript.

## References

[1] Armstrong A.W., Read C. (2020). Pathophysiology, clinical presentation, and treatment of psoriasis: A review. JAMA.

[2] Samotij D., Nedoszytko B., Bartosińska J., Batycka-Baran A., Czajkowski R., Dobrucki I.T., Dobrucki L.W., Górecka-Sokołowska M., Janaszak-Jasienicka A., Krasowska D. (2020). Pathogenesis of psoriasis in the “omic” era. Part I. Epidemiology, clinical manifestation, immunological and neuroendocrine disturbances. Postep. Dermatol. Alergol..

[3] Jiang Y., Chen Y., Yu Q., Shi Y. (2023). Biologic and Small-Molecule Therapies for Moderate-to-Severe Psoriasis: Focus on Psoriasis Comorbidities. BioDrugs.

[4] Peng Y., Tao Y., Zhang Y., Wang J., Yang J., Wang Y. (2023). CD25: A potential tumor therapeutic target. Int. J. Cancer.

[5] Panackel C., Mathew J.F., Fawas N.M., Jacob M. (2022). Immunosuppressive Drugs in Liver Transplant: An Insight. J. Clin. Exp. Hepatol..

[6] Kanda N., Hoashi T., Saeki H. (2021). The Defect in Regulatory T Cells in Psoriasis and Therapeutic Approaches. J. Clin. Med..

[7] Gorabi A.M., Hajighasemi S., Kiaie N., Gheibi Hayat S.M., Jamialahmadi T., Johnston T.P., Sahebkar A. (2020). The pivotal role of CD69 in autoimmunity. J. Autoimmun..

[8] Adamczyk M., Bartosińska J., Raczkiewicz D., Michalak-Stoma A., Krasowska D. (2023). The Impact of Biologic Treatment on PD-1/PD-L1 Pathway Disturbances in Psoriasis. J. Clin. Med..

[9] Mrowietz U., Zhu K., Christophers E. (2000). Treatment of severe psoriasis with anti-CD25 monoclonal antibodies. Arch. Dermatol..

[10] Fan Z., Li L., Wang X., Miao G. (2020). Dysfunction of regulatory T cells mediated by AKT-FOXO1 signaling pathway occurs during the development of psoriasis. Int. J. Clin. Exp. Pathol..

[11] Li B., Lei J., Yang L., Gao C., Dang E., Cao T., Xue K., Zhuang Y., Shao S., Zhi D. (2019). Dysregulation of Akt-FOXO1 Pathway Leads to Dysfunction of Regulatory T Cells in Patients with Psoriasis. J. Investig. Dermatol..

[12] Su Q.Y., Zhang S.X., Yang L., Luo J., Li X.F., Zhang J.Q., Zhang Y., Liu J.Q., Shi L. (2023). Peripheral Treg Levels and Transforming Growth Factor-β (TGFβ) in Patients with Psoriatic Arthritis: A Systematic Review Meta-Analysis. Adv. Ther..

[13] Mehta H., Mashiko S., Angsana J., Rubio M., Hsieh Y.M., Maari C., Reich K., Blauvelt A., Bissonnette R., Muñoz-Elías E.J. (2021). Differential Changes in Inflammatory Mononuclear Phagocyte and T-Cell Profiles within Psoriatic Skin during Treatment with Guselkumab vs. Secukinumab. J. Investig. Dermatol..

[14] Quaglino P., Ortoncelli M., Comessatti A., Ponti R., Novelli M., Bergallo M., Costa C., Cicchelli S., Savoia P., Bernengo M.G. (2009). Circulating CD4+CD25 bright FOXP3+ T cells are up-regulated by biological therapies and correlate with the clinical response in psoriasis patients. Dermatology.

[15] De Pità O., Ruffelli M., Cadoni S., Frezzolini A., Biava G.F., Simom R., Bottari V., De Sanctis G. (1996). Psoriasis: Comparison of immunological markers in patients with acute and remission phase. J. Dermatol. Sci..

[16] Porto Ferreira C., Gomes da Silva A., Martins C.J., Da-Cruz A.M. (2011). CD25^+^CD8^+^, CLA^+^CD4^+^, CD11a^+^ CD4^+^, and CD11a^+^ CD8^+^ T cell counts are elevated in the blood of Brazilian patients with chronic plaque psoriasis. Actas Dermosifiliogr..

[17] Economidou J., Barkis J., Demetriou Z., Avgerinou G., Psarra K., Degiannis D., Vareltzidis A., Katsambas A. (1999). Effects of cyclosporin A on immune activation markers in patients with active psoriasis. Dermatology.

[18] Langewouters A.M., Bovenschen H.J., De jong E.M., Van Erp P.E., Van De Kerkhof P.C. (2007). The effect of topical corticosteroids in combination with alefacept on circulating T-cell subsets in psoriasis. J. Dermatol. Treat..

[19] Reddy M., Torres G., McCormick T., Marano C., Cooper K., Yeilding N., Wang Y., Pendley C., Prabhakar U., Wong J. (2010). Positive treatment effects of ustekinumab in psoriasis: Analysis of lesional and systemic parameters. J. Dermatol..

[20] Gertel S., Polachek A., Furer V., Levartovsky D., Sidis H., Pel S., Paran D., Elkayam O. (2022). T cell functions of psoriatic arthritis patients are regulated differently by TNF, IL-17A and IL-6 receptor blockades in vitro. Clin. Exp. Rheumatol..

[21] Khalil S., Bardawil T., Kurban M., Abbas O. (2020). Tissue-resident memory T cells in the skin. Inflamm. Res..

[22] Cibrian D., Saiz M.L., de la Fuente H., Sánchez-Díaz R., Moreno-Gonzalo O., Jorge I., Ferrarini A., Vázquez J., Punzón C., Fresno M. (2016). CD69 controls the uptake of L-tryptophan through LAT1-CD98 and AhR-dependent secretion of IL-22 in psoriasis. Nat. Immunol..

[23] Kim J., Bissonnette R., Lee J., Correa da Rosa J., Suárez-Fariñas M., Lowes M.A., Krueger J.G. (2016). The Spectrum of Mild to Severe Psoriasis Vulgaris Is Defined by a Common Activation of IL-17 Pathway Genes, but with Key Differences in Immune Regulatory Genes. J. Investig. Dermatol..

[24] Diani M., Casciano F., Marongiu L., Longhi M., Altomare A., Pigatto P.D., Secchiero P., Gambari R., Banfi G., Manfredi A.A. (2019). Increased frequency of activated CD8^+^ T cell effectors in patients with psoriatic arthritis. Sci. Rep..

[25] Cameron A.L., Kirby B., Griffiths C.E. (2003). Circulating natural killer cells in psoriasis. Br. J. Dermatol..

[26] Lankford K.V., Mosunjacm M., Hillyer C.D. (2000). Effects of UVB radiation on cytokine generation, cell adhesion molecules, and cell activation markers in T-lymphocytes and peripheral blood HPCs. Transfusion.

[27] Gaál J., Lakos G., Szodoray P., Kiss J., Horváth I., Horkay E., Nagy G., Szegedi A. (2009). Immunological and clinical effects of alphacalcidol in patients with psoriatic arthropathy: Results of an open, follow-up pilot study. Acta Derm. Venereol..

[28] Haider A.S., Lowes M.A., Gardner H., Bandaru R., Darabi K., Chamian F., Kikuchi T., Gilleaudeau P., Whalen M.S., Cardinale I. (2007). Novel insight into the agonistic mechanism of alefacept in vivo: Differentially expressed genes may serve as biomarkers of response in psoriasis patients. J. Immunol..

[29] Rubant S.A., Ludwig R.J., Diehl S., Hardt K., Kaufmann R., Pfeilschifter J.M., Boehncke W.H. (2008). Dimethylfumarate reduces leukocyte rolling in vivo through modulation of adhesion molecule expression. J. Investig. Dermatol..

[30] Torres-Alvarez B., Castanedo-Cazares J.P., Fuentes-Ahumada C., Moncada B. (2007). The effect of methotrexate on the expression of cell adhesion molecules and activation molecule CD69 in psoriasis. J. Eur. Acad. Dermatol. Venereol..

[31] Sigmundsdottir H., Gudjonsson J.E., Valdimarsson H. (2003). The effects of ultraviolet B treatment on the expression of adhesion molecules by circulating T lymphocytes in psoriasis. Br. J. Dermatol..

[32] Owczarczyk-Saczonek A., Kasprowicz-Furmańczyk M., Czerwińska J., Krajewska-Włodarczyk M., Placek W. (2022). The effect of therapy on TRM in psoriatic lesions. Adv. Dermatol. Allergol./Postępy Dermatol. Alergologii.

